# Case report: Hematologic malignancies concomitant diagnosis of hairy cell leukemia and chronic lymphocytic leukemia: A rare association

**DOI:** 10.3389/fonc.2022.1069977

**Published:** 2022-12-05

**Authors:** Luciana Valvano, Fiorella D’Auria, Vitina Grieco, Teodora Statuto, Filomena Nozza, Giuseppe Pietrantuono, Oreste Villani, Giovanni D’Arena, Daniela Lamorte

**Affiliations:** ^1^ Laboratory of Clinical and Advanced Diagnostics, Centro di Riferimento Oncologico della Basilicata (IRCCS CROB), Rionero in Vulture, Italy; ^2^ Laboratory of Clinical Pathology, Centro di Riferimento Oncologico della Basilicata (IRCCS CROB), Rionero in Vulture, Italy; ^3^ Hematology and Stem Cell Transplantation Unit, Centro di Riferimento Oncologico della Basilicata (IRCCS CROB), Rionero in Vulture, Italy; ^4^ Hematology, P. O. S. Luca, ASL, Salerno, Italy; ^5^ Laboratory of Preclinical and Translational Research, Centro di Riferimento Oncologico della Basilicata (IRCCS CROB), Rionero in Vulture, Italy

**Keywords:** hairy cell leukemia, chronic lymphocytic leukemia, flow cytometry, BRAF V600E mutation, droplet digital PCR

## Abstract

A case of concomitant hairy cell leukemia (HCL) and chronic lymphocytic leukemia (CLL) in a 50- year-old man was reported. Flow cytometry and droplet digital PCR (ddPCR) were used to detect the B-Raf proto-oncogene (BRAF) V600E mutation. The HCL population was the predominant component. The patient was first treated with cladribine and then with rituximab and achieved HCL partial remission. Importantly, the high sensitivity of our flow cytometric approach allowed the detection of a small population “P3,” in addition to the typical HCL and CLL clones. The P3 clone changed over time, from an HCL-like to a CLL-like immunophenotype. This case is added to the few other cases of synchronous HCL and CLL already reported in the literature and underlines the importance of analyzing chronic lymphoproliferative disorders by highly sensitive diagnostic techniques, like the multicolor flow cytometry and ddPCR, to evaluate the possible association between HCL and CLL at diagnosis.

## Introduction

Hairy cell leukemia (HCL) is a rare neoplasm representing 2% of all lymphoid leukemia (1). The median age at diagnosis is 55 years, and it predominantly affects men. Typically, HCL patients show cytopenias, splenomegaly, a low percentage of circulating hairy cells, and diffuse leukemic bone marrow infiltration. The B-RAf proto oncogene (BRAF) V600E point mutation occurs in 97% of HCL patients and is responsible for the typical “hairy” appearance of the HCL cells (1). This mutation constitutively activates the RAS-RAF-MEK-ERK signaling pathway, inducing cellular proliferation and survival. Hairy cells have a typical pattern of B-cell antigen expression (CD19+, CD20+, and CD22+) with the coexpression of CD11c, CD25, and CD103. Although the association of HCL with other neoplasms is well known, the simultaneous diagnosis of HCL and other tumors is very rare. A case of concomitant diagnosis of HCL and B-cell chronic lymphocytic leukemia (CLL) was reported and compared with other literature-reported ones.

## Case presentation

**Figure 1:**
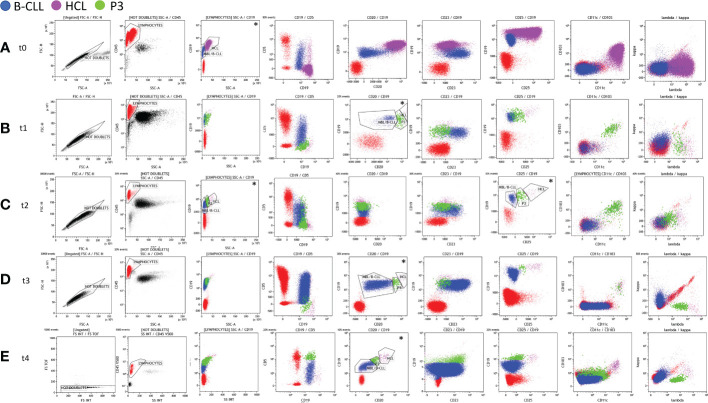
Immunophenotyping analysis of the bone marrow (BM) aspirate (t0, t1, and t2) and peripheral blood (PB) (t3 and t4) carried out at diagnosis (t0 – panel **A**), at first evaluation after cladribine therapy (t1 – panel **B**), and after rituximab treatment (t2 – panel **C**, t3 – panel **D**, and t4 – panel **E**).Any debris, dead cells, and clumps or doublets were excluded using forward scatter (FSC)-height (FSC-H) by FSC-area (FSC-A) parameters (black gate, “Not Doublets”). CD45+ lymphocytes (red gate) were gated on CD45 *vs*. the side scatter (SSC) dot plot on the Not Doublets gate. Hairy cell leukemia (HCL), monoclonal B-cell lymphocytosis (MBL)/B-cell chronic lymphocytic leukemia (B-CLL), and P3 populations (fuchsia, blue, and green gates, respectively) were identified from the lymphocyte gate. The expression of some specific markers (CD19, CD20, and CD25) and the physical parameter of SSCs were exploited to identify the three clonal populations (see the dot plots marked with *). At t0 **(A)**, the HCL and MBL/B-CLL clones were identified by the different expression of CD19 *vs*. SSC. At t1 **(B)**, t3 **(D)**, and t4 **(E)**, the three B-cell clones were identified by coexpression at the variable intensity of the CD19 and CD20 markers. At t2 **(C)**, following treatment with rituximab, the gating strategy was based on the different intensities of the expression of CD19 *vs*. CD25 and/or CD19 *vs*. SSC.

A 50-year-old man was referred to our center because of fatigue for approximately 2 months, neutropenia, anemia, and thrombocytopenia (**Table 1**). Pale skin and splenomegaly were also found at physical examination. A peripheral blood (PB) smear showed lymphocytes (90%), neutrophils (10%), and 0% monocytes. Two distinct monoclonal B-cell populations were detected by flow cytometry immunophenotypic analyses. The predominant population, approximately 66% of the white blood cell (WBC) count, was consistent with HCL: CD19^+high^, CD20^+high^, CD5^-^, CD23^-^, CD43^-^, CD10^-^, CD103^+^, CD25^+^, CD11c^+^, CD79b^+^, CD200^+^, FMC7^+^, CD22^+high^, and sIgλ^+high^. A smaller population (approximately 17%) showed the CD19^+intermediate^, CD20^+low^, CD5^+intermediate^, CD23+, CD43+, CD10-, CD103-, CD25-, CD11c-, CD79b-, CD200+, FMC7+/-, CD22+^low^, and sIgκ+^low^ immunophenotype, consistent with monoclonal B-cell lymphocytosis (MBL), typical B-CLL-like (**Table 2**, **Figure 1A**). The hairy cells were counted as monocytes by automated blood cell counters (**Table 1**). There were 90% of hairy cells with a minimal localization of small-sized lymphocytes found upon morphological examination of the bone marrow (BM). Two distinct monoclonal B-cell populations, consistent with HCL and MBL, were also found in the BM by flow cytometry analyses. The BRAF V600E mutation evaluated by droplet digital PCR (ddPCR) was found with 38% marrow involvement. Fluorescence in situ hybridization (FISH) analysis performed on fixed nuclei using the commercially available Vysis CLL FISH Probe Kit showed the deletion of chromosome 13 and the presence of wild-type Tp53 and ATM genes. Finally, the diagnosis of a composite predominant HCL with a minor clone of MBL CLL-like was made.

**Table 1:** Complete blood cell count of our case at diagnosis (t0) and after therapy (t1–t4).

CBC	t_0_	t_1_	t_2_	t_3_	t_4_
Hemoglobin	8.1 g/dl (L)	13.5 g/dl (N)	15.8 g/dl (N)	15.5 g/dl (N)	15.8 g/dl (N)
Absolute neutrophil count	0.5 × 10^3^/µl (L)	1 × 10^3^/µl (L)	3.4 × 10^3^/µl (N)	1.3 × 10^3^/µl (L)	0.8 × 10^3^/µl (L)
Absolute lymphocyte count	4.8 × 10^3^/µl (N)	1.5 × 10^3^/µl (N)	0.7 × 10^3^/µl (L)	2.4 × 10^3^/µl (N)	5.8 × 10^3^/µl (H)
Absolute monocyte count	13.9 × 10^3^/µl (H)	0 × 10^3^/µl (L)	0 × 10^3^/µl (L)	0.1 × 10^3^/µl (L)	0.1 × 10^3^/µl (L)
Platelet count	56 × 10^3^/µl (L)	128 × 10^3^/µl (L)	169 × 10^3^/µl (N)	131 × 10^3^/µl (N)	107 × 10^3^/µl (L)

N, normal value; L, low value; H, high value; and CBC, complete blood cell.

The high value of the monocyte count at t0 refers to the hairy cell count obtained by automated blood cell counters.

**Table 2:** Percentage of the three clonal populations detected by flow cytometric analysis on white blood cells (WBCs) and of the BRAF V600E mutation performed on the peripheral blood (PB) and/or bone marrow (BM) at diagnosis (t0) and after treatment (t1–t4).

Time	Clone	% on WBC	% BRAF V600E
t0 (PB)	HCL	65.85	
	CLL	17.32	
	P3	n.i.	
t0 (BM)	HCL	55.3	38
	CLL	19	
	P3	n.i.	
t1 (BM)	HCL	0.91	1
	CLL	15.68	
	P3	0.94	
t2 (BM)	HCL	0.69	2.3
	CLL	11.69	
	P3	1.54	
t3 (PB)	HCL	0.24	0.25
	CLL	24.9	
	P3	0.23	
t4 (PB)	HCL	0.85	0.65
	CLL	34.8	
	P3	1.29	

PB, peripheral blood; BM, bone marrow; WBCs, white blood cells; and n.i., not identified.

The patient was treated with cladribine after informed consent was given. At +3 months after the diagnosis (t1), he achieved partial remission (PR) with moderate neutropenia and thrombocytopenia and the resolution of anemia (**Table 1**). The immunophenotyping of BM aspirate revealed an HCL population reduction to 0.9%, while the CLL population was 16% among WBCs (**Table 2**, **Figure 1B**). Likewise, the persistence of the BRAF V600E mutation (1%) was described (**Table 2**). For that reason, additional four weekly cycles followed by another four biweekly cycles of rituximab were also given, followed by a watch-and-wait approach on the PR status. Either cladribine or rituximab was well tolerated without relevant side effects.

The persistence of the two pathological B-cell populations (HCL and MBL, both without CD20 surface expression) was detected in the BM 1 month after the end of rituximab treatment (t2) (**Table 2**, **Figure 1C**). The BRAF V600E mutation was still detectable (**Table 2**). At +8 months (t3), the flow cytometric examination of the PB sample again evidenced the presence of both HCL and MBL populations, both positive for CD20 (**Table 2**, **Figure 1D**).

At the last follow-up (+27 months: t4), the percentage of the BRAF V600E mutation found on the PB was 0.65% and the immunophenotyping analysis of the PB confirmed the presence of both HCL and CLL populations (**Table 2**, **Figure 1E**). In this case, indeed, the number of clonal B cells was found higher than 5.000/µl, consistent with the diagnosis of CLL. Moreover, a third clone (P3: 1.3% of WBCs) was identified owing to the particular expression (intermediate intensity) of the λ light chain in a portion of CD5+ B cells (**Table 2**, **Figure 1E**). The P3 population showed an MBL/B-CLL phenotype with a characteristic intensity of CD20 and CD19 markers that was used for the gating strategy. In particular, P3 expressed CD19^+intermediate^, CD20^+intermediate^, CD23^+^, CD5^+high^, CD25^-^, CD11c^-^, CD43^+^, CD103^-^, CD79b^+low^, FMC7^+low^, sIgĸ^-^, and sIgλ^+intermediate^ immunophenotype (**Figure 1E**). Based on this result, we researched P3 analyzing the previous flow cytometric files. **Figure 1** shows the gating strategy and the immunophenotype of the three populations at different time points. P3 was absent at diagnosis (t0) and appeared in post-therapy samples (**Figure 1**). In particular, we found a P3 clone (0.94% of WBCs) in t1 at first evaluation after cladribrine treatment (**Figure 1B**, **Table 2**). It showed an HCL-like immunophenotype: CD19^+ intermediate^, CD20^+intermediate^, CD23^-^, CD5^-^, CD25^+intermediate^, CD11c^+^, CD103^+^, sIgĸ^-^, and sIgλ^+intermediate^ (**Figure 1B**). For this reason, we hypothesized that the P3 population may have originated from the HCL clone. In t2 (+1 month after the end of rituximab treatment), P3 was 1.5% of WBCs and it showed the same immunophenotype of t1 except for CD20 that was absent (**Table 2**, **Figure 1C**). P3 was also identified in t3 (8 months after the end of rituximab treatment) representing 0.23% of WBCs and showing the same immunophenotype of t1 (**Table 2**, **Figure 1D**). Finally, we established to monitor the patient with a watch-and-wait approach until disease progression (HCL or CLL diseases).

## Discussion

Although the coexistence of HCL and second tumors, including other hematological malignancies, has been already described, the simultaneous diagnosis of HCL and CLL is considered quite rare (2). From 2002 to date, to the best of our knowledge, only 11 cases of synchronous diagnosis of HCL and CLL have been published (**Table 3**) (2–4, 6–10). A new case of concomitant diagnosis of HCL and CLL has been reported here and compared with literature-reported ones. All patients were men with pancytopenia and splenomegaly. In our case, the HCL population was the predominant component in both the PB and BM at diagnosis. Only two other cases (2 and 10) showed similar data, while in all other cases, the CLL population was predominant in the PB and the HCL population was predominant in the BM (**Table 3**) (2, 8). Since HCL cells are typically rare in the PB, the use of highly sensitive diagnostic techniques, including multicolor flow cytometry and allele‐specific PCR, were useful for their detection. Different molecular techniques, like ddPCR and next-generation sequencing, are recently introduced as sensitive approaches for assessing the BRAF V600E mutation in HCL patients. In our case, we analyzed both the PB and BM aspirate samples by flow cytometry using a stain–lyse-no wash technique and a comprehensive seven-color antibody panel (FITC/PE/PerCP-Cy5.5/PE-Cy7/APC/APC-H7/V500 fluorescent conjugates) and evaluated the BRAF V600E mutation by ddPCR. The high sensitivity of our flow cytometric approach allowed the detection of a small population “P3,” in addition to the typical HCL and CLL clones. The P3 population was absent at diagnosis and it appeared in post-therapy samples (**Figure 1**). P3 showed an immunophenotype intermediate between HCL and CLL although its features changed over time (**Figure 1**). In particular, at the first evaluation (t1), P3 showed an HCL-like immunophenotype and it differed from the HCL clone mainly for the intensity expression of CD19, CD20, CD25, and the λ light chain. For this reason, we hypothesized that the P3 population may have originated from the HCL clone. From t1 to t3, the P3 immunophenotype remained the same with the only exception of CD20 that became negative in t2 following rituximab treatment. At the last follow-up (t4), the P3 immunophenotype changed assuming predominantly CLL-like features. In particular, it expressed CD23^+^, CD5^+high^ without CD25, CD11c, and CD103 (**Figure 1**). However, unlike CLL, it was positive for the λ light chain.

**Table 3:** Clinical characteristics of simultaneous diagnosis of hairy cell leukemia (HCL) and B-cell chronic lymphocytic leukemia (CLL) cases.

Case	Age	Sex	Treatment	Outcome	Predominant B-cell population in PB	Predominant B-cell population in BM	BRAF V600E mutation	Secondary tumors	Reference no.
1	50	M	I. Cladribine II. Rituximab	HCL partial remission, stable CLL	HCL	HCL	Yes (ddPCR)		Our case
2	59	M	I. DCF II. 2-CDA	HCL in remission, stable CLL	HCL	HCL	Not reported		(2)
3	72	M	I. Splenectomy II. Trametinib and dabrafenib	Not reported	Not reported	HCL	Yes (immunohistochemistry)		(3)
4	77	M	I. Cladribine	HCL in remission, CLL immunophenotype occupying 5% of BM	HCL	CLL	Yes (NGS)		(4)
5	75	M	Patient declined therapy	Not reported	CLL	CLL/SLL	Not reported		(5)
6	69	M	Rituximab	HCL in remission, stable CLL	CLL (CD19+/CD20-/CD5+/CD23+/dim surface κ+/CD25-/CD103-)	HCL	Not reported	Thyroid cancer and multiple pulmonary and pleural nodes	(6)
7	43	M	Cladribine	Worsening lymphadenopathy	CLL (CD19+/CD20+/CD5+/CD23+/moderate-density λ+)	HCL	Not reported		(6)
8	79	M	Treated for lung cancer	Transfer to another facility	CLL	HCL	Not reported		(6)
9	83	M	I. Cholambucil and prednisone II. 2-CDA III. Fludarabine, cytoxan, and G-CSF IV. Rituximab	Stable HCL, CLL in remission	CLL		Not reported		(7)
10	54	M	I. Cladribine II. Rituximab	HCL in remission, residual CLL	HCL	HCL	Yes (allele-specific PCR)		(8)
11	63	M	I. 2-CDA (1 year after diagnosis)	Decrease of HCL population; stable CLL	CLL	HCL	Not reported		(9)
12	72	M	Pentostatin and rituximab	Residual HCL, CLL in remission	Not reported	HCL	Not reported		(10)

M, male; DCF, deoxycoformycin; 2-CDA, 2-chloroadenosine; G-CSF, granulocyte colony-stimulating factor; HCL, hairy cell leukemia; B-CLL, B-cell chronic lymphocytic leukemia; BRAF, B-Raf proto oncogene; ddPCR, droplet digital PCR; and NGS = next-generation sequencing.

Zhang et al. previously described a case of composite HCL and CLL showing three distinct B-cell populations at diagnosis (case 4, **Table 3**) (4). The third population expressed CD11c and bright CD20 and lacked CD25 and CD103. Unlike what was described in our case, the authors did not tell of any immunophenotyping change in their third population but described a low-variant allele frequency for the BRAF V600E mutation in their P3 population at diagnosis. Unfortunately, we could not sort out our P3 clone so we could not evaluate the possible presence of the BRAF V600E mutation. However, although the BRAF V600E mutation represents the genetic cause of HCL and it allows the differential diagnosis between the HCL and other B-cell neoplasms, including the HCL variant and splenic marginal zone lymphoma; it was evaluated only in two other cases reported in literature. In particular, Liptrot et al. (case 10) combined a six-color immunophenotypic analysis with an allele-specific PCR approach to detect the HCL cells and to assess the BRAF V600E mutation, and Francischetti and Calvo (case 3) detected the said mutation by immunohistochemistry (**Table 3**) (3, 8). Only case 10 received the same treatment as our patient (cladribine and rituximab), achieving HCL remission but with the persistence of the CLL clone (8). However, while no evidence of the BRAF V600E mutation was detected in case 10 after treatment, in our case, the same mutation was still detectable (0.65%) by ddPCR, 27 months after the end of rituximab treatment. Cases 2, 4, and 6 also described patients who achieved HCL remission but with the persistence of CLL after treatment, although they were differently treated (**Table 3**) (2, 4, 6). Cases 9 and 12 were the only two cases that reported CLL remission and the persistence of a small HCL population in the BM after different courses of therapy (7, 10). However, although the large availability of new therapeutic agents has improved the survival of HCL patients, HCL is often associated with an increased risk of second tumor development, including lymphomas, chronic lymphocytic leukemia/small lymphocytic lymphoma (CLL/SLL), chronic myelogenous leukemia (CML), lymphoma, and thyroid cancer (6). Among all cases of the simultaneous diagnosis of HCL and CLL, only one patient (case 6) developed second malignancies (**Table 3**). In particular, case 6 developed papillary thyroid cancer and multiple pulmonary/pleural nodes 51 months after diagnosis and he passed away. Additionally, case 8 described a patient with the concurrent diagnosis of HCL, CLL, and a third neoplasm, specifically lung adenocarcinoma (**Table 3**). In this case, the patient was treated for lung cancer first and he was lost to follow-up. Finally, a particular case of composite HCL and CLL was case 11: a patient with simultaneous B- and T-cell disorders at diagnosis (9). In particular, the patient showed three clones, namely, HCL, CLL, and CD4^++^/CD8^+^ T-cell large granular lymphocytosis. A decrease in the percentage of HCL subpopulation and a stable percentage of CLL cells were reported after the 2-CDA therapy.

All patients were treated for HCL or for their simultaneous neoplasms, except for case 5 because the patient declined all kinds of therapy (5).

In conclusion, our case is added to the few other cases of synchronous HCL and CLL already reported in the literature and underlines the importance of analyzing chronic lymphoproliferative disorders by highly sensitive diagnostic techniques, like the multicolor flow cytometry and ddPCR, to evaluate the possible association between HCL and CLL at diagnosis and to monitor minimal residual disease after therapy.

## Data availability statement

The original contributions presented in the study are included in the article. Further inquiries can be directed to the corresponding authors.

## Ethics statement

Ethical review and approval was not required for the study on human participants in accordance with the local legislation and institutional requirements. Written informed consent was obtained from the individual for the publication of any potentially identifiable images or data included in this article.

## Author contributions

LV, FD’A and TS performed flow cytometric analyses and analyzed the data. VG performed droplet digital PCR to detect the BRAF V600E mutation. FN performed the FISH analysis. GP and OV monitored the patient in all phases of the disease. DL performed literature search and wrote the original draft, and GD’A revised the manuscript. All authors contributed to the article and approved the submitted version.

## Funding

This work was supported by the Italian Ministry of Health - Ricerca Corrente 2022.

## Acknowledgments

We thank the patient and his family for giving consent to this case report.

## Conflict of interest

The authors declare that the research was conducted in the absence of any commercial or financial relationships that could be construed as a potential conflict of interest.

## Publisher’s note

All claims expressed in this article are solely those of the authors and do not necessarily represent those of their affiliated organizations, or those of the publisher, the editors and the reviewers. Any product that may be evaluated in this article, or claim that may be made by its manufacturer, is not guaranteed or endorsed by the publisher.
